# Camouflage versus running performance as strategies against predation in a lizard inhabiting different habitats

**DOI:** 10.1002/ece3.8374

**Published:** 2021-11-20

**Authors:** Lixia Wan, Zhenxia Liu, Tao Wang, Minglu Yang, Jiasheng Li, Hui Sun, Chenkai Niu, Wei Zhao, Yuanting Jin

**Affiliations:** ^1^ College of Life Sciences Northwest Normal University Lanzhou China; ^2^ College of Life Sciences China Jiliang University Hangzhou China; ^3^ College of life sciences and medicine Zhejiang Sci‐Tech University Hangzhou China; ^4^ College of Life Sciences Lanzhou University Lanzhou China

**Keywords:** anti‐predation, camouflage, color variation, reptile, running speed

## Abstract

Running speed and camouflage are associated with the foraging and anti‐predator abilities of animals. The toad‐headed lizard, *Phrynocephalus versicolor*, has evolved a darker dorsal color in melanistic habitats and maintained a lighter color in adjacent, non‐melanistic habitats. We test the hypothesis that lizards have weaker running speed on well‐matching backgrounds than on less matching backgrounds. We used lizard models to compare the predation pressure, while the running speed of dark and light lizards were compared in field tunnels using a video recording method. Our results indicated that both the dark lizards in melanistic Heishankou (HSK) and the light lizards in non‐melanistic Guazhou (GZ) face lower predation pressure than potential color‐background unmatched lizards. The light lizards have a potentially higher running speed than darker lizards in melanistic habitats, which implies that substrate color matching populations with benefits of camouflage might have lower anti‐predation pressure, and the costs of investment in melanin production may reduce running capacity.

## Graphical abstract

The lizards with substrate matching dorsal color have lower predation pressure than substrate unmatched lizards. The dark lizards with benefits of camouflage lowed the running speed in melanistic habitat.

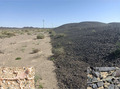

## 1 INTRODUCTION

Animals employ various strategies to cope with predation or anti‐predation pressure through morphological and/or behavioral changes (Bauwens & Thoen, 1981; Duchateau et al., 2007; Ge et al., 2011; Vermeij, 1994; Watson et al., 2012). Some animals employ camouflage successfully to strengthen the efficiency of predator–prey interactions (Stuart‐Fox et al., 2006; Tong et al., 2016, 2019), while others adapt to it by improving their running abilities (Cooper, 2010; Husak, 2006a; Kravchuk & Watson, 2020).

Camouflage is key for animals to survive in different color backgrounds (Johansson & Nilsson‐Örtman, 2013; Manríquez et al., 2008; Merilaita & Stevens, 2011). Because the visual background is the main basis of camouflage, it is crucial for various species to adapt their body coloration to the surrounding substrate, including fish (Kelley et al., 2017; Kjernsmo & Merilaita, 2012), amphibians (Polo‐Cavia et al., 2016; Rabbani et al., 2015), reptiles (Hamilton et al., 2008; Kravchuk & Watson, 2020; Krohn & Rosenblum, 2016; Marshall et al., 2016; Merilaita & Lind, 2005), birds (Lovell et al., 2013), and insects (Batty, 2008; Kang et al., 2015). The camouflage strategy not only benefits the prey by defending it against predators, but also helps predators capture the prey more efficiently (Cadena et al., 2017; Cook et al., 2012; Edelaar et al., 2017).

Running ability is also of primary importance for animals to adapt to predation or anti‐predation pressure (Cooper, 2010; Husak, 2006a, 2006b). Rapid flight when a predator is detected is one of the main anti‐predation strategies in lizards (Martín & López, 2000), and consequently, survival increases with higher sprint speed (Irschick & Meyers, 2007). High locomotor performance helps predators chase prey, helps prey escape predators, and even improves competitiveness (Higham et al., 2011).

Natural selection favors anti‐predatory and antiparasitic strategies to improve animal's fitness, and animals eliciting an immune response should reduce their sprint capacity, as has been shown in the lizard *Psammodromus algirus* (Zamora‐Camacho et al., 2015). It indicates the trade‐off between the energy cost of sprint speed and immune response (Zamora‐Camacho et al., 2015). Both the running traits and melanin synthesis need proteins and are energetically costly (Hill et al., 2006; Moreno Rueda, 2020; Zamora‐Camacho et al., 2015). Moreover, investment in melanism may imply a cost in terms of oxidative stress or reduced immunocompetence (Ducrest et al., 2008). Therefore, to cope with anti‐predation pressure, we predict lizard will trade off camouflage and running speed. More investment in early crypsis may also mean there is less energy to invest in running ability.

**FIGURE 1:**
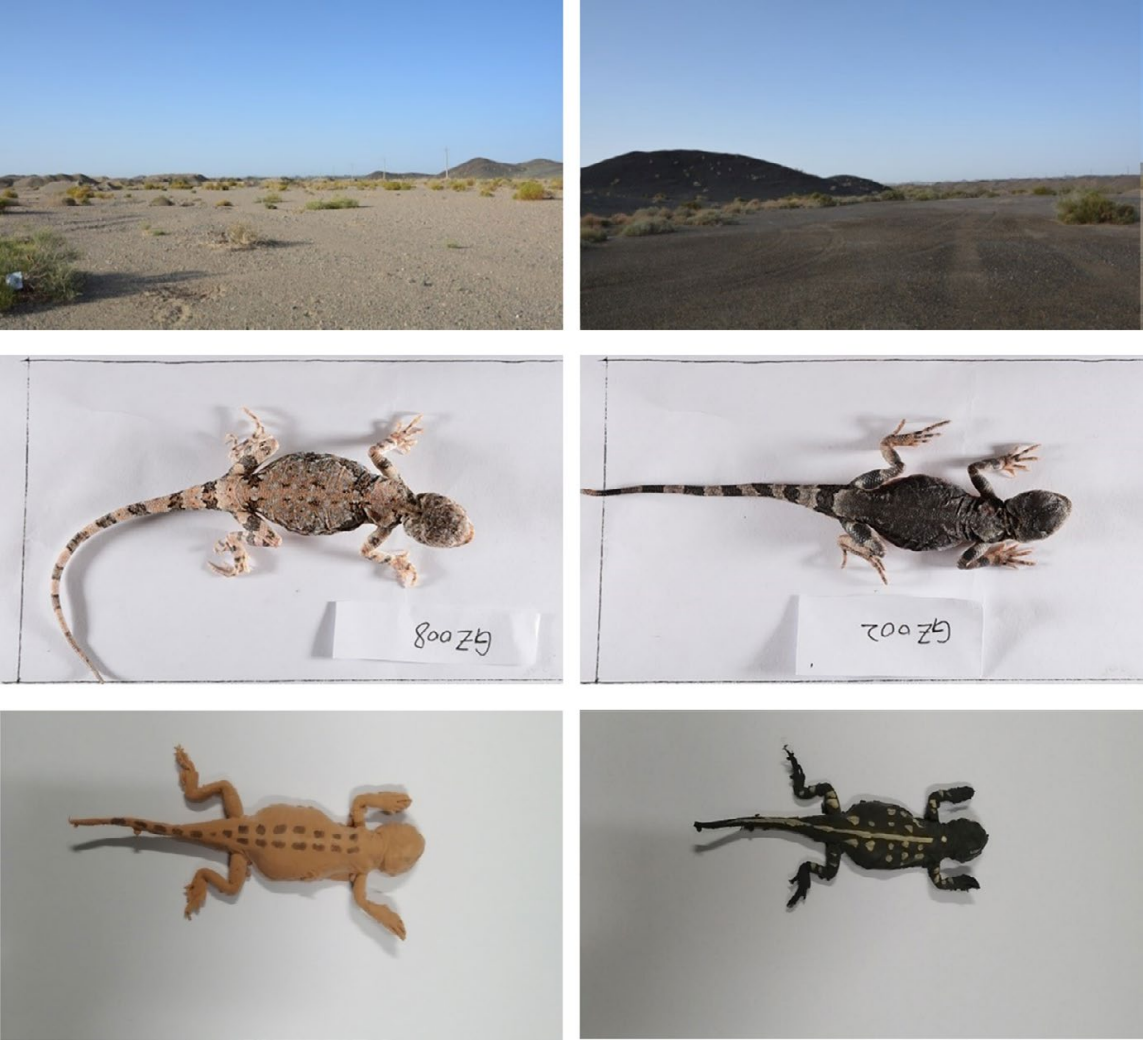
Typical sampling and habitats of *Phrynocephalus versicolor*. The non‐melanistic habitat, light adult lizard and lizard model (left), the melanistic habitat, dark adult lizard and lizard model (right)

The toad‐headed lizard, *Phrynocephalus versicolor*, widely inhabits the deserts and semideserts endemic in eastern Xinjiang, western Inner Mongolia, western Gansu, and Ningxia province in China (Zhao & Zhou, 1999). The HSK is a typical natural “blackening” area (from the geomorphology, it is a natural blackening with a long history, rather than recently formed by man‐made interference). We observed that *P*. *versicolor* has evolved a darker dorsal color in the melanistic, mountainous area of HSK, but has evolved a lighter dorsal color in other known distributions of the species, including the adjacent non‐melanistic Gobi habitats in GZ (see Figure 1). Studies have shown that there are many factors that cause lizard body color variation. The lizards may have evolved darker dorsal color because pressure of predation selects for cryptic coloration in melanic substrate color (Husak et al., 2006; Reguera et al., 2014). Other studies have suggested that altitude has an effect on the body color variation of lizards, and darkness coloration might have evolved in response to adverse conditions (low temperature and high UV radiation) at high altitudes (Moreno‐Rueda et al., 2019; Reguera et al., 2014). Although the elevation of HSK (1602m) is higher than nearby non‐melanic desert (1412 m), and HSK has many high mountains covered with black stones. Non‐melanic populations from large areas with variable elevation‐associated environmental conditions show similar light dorsal color, while the melanic population in HSK exhibits dark dorsal color in high altitude as well, indicating that substrate color rather than elevation is the key influence on color variation (Tong et al., 2019). This camouflage variation is mainly due to long‐term genetic adaptation rather than phenotypic plasticity (Tong et al., 2019).

Moreover, lizard models were used to study the predation pressure on lizards with different dorsal colors. Some studies proposed that lizards with less conspicuous coloration did not flee immediately to minimize their exposure and costs of fleeing (Martín et al., 2009). We assumed that the running ability of the darker HSK lizards correlated with their anti‐predator capacity and/or crypsis costs. Alternatively, the camouflage populations would fulfill anti‐predator requirements primarily through background color matching, and/or make investment in crypsis more costly, which would decrease the selection pressure on running speed (Miller et al., 2010). To test these hypotheses, we compared the running speed of dark and light lizards in melanistic and non‐melanistic habitats, using the field racetrack recording method.

## 2 MATERIALS AND METHODS

### 2.1 Sampling

The ground of the HSK area in Liuyuan town, Gansu Province, is quite different from that of the nearby Gobi Desert of GZ. The former is much darker, and its surface matrix is mainly composed of exposed black stones, rich in iron elements; the latter is lighter and mainly consists of light‐colored yellow sandstone (Figure 1). From August 10 to August 13, 2019, a total of 64 adult lizards, including 43 dark lizards (13 males, 30 females) and 21 light lizards (8 males and 13 females), were collected from melanistic HSK region and its adjacent non‐melanistic region (GZ) for examination of their running abilities (Figure 1). The lizards were captured and an individual identity code was written on the abdomen using a marker pen. Geographical information of the sampling locations is shown in online supplementary table S1.

### 2.2 Running speed

Running speed was determined using field racing tunnels (120 × 5.7 × 4.8 cm), with a scale on the edge of it, placed on localities comprising both natural melanistic and non‐melanistic substrates. The running speed in a continuous running period without stopping was used in the following analyses. We recorded lizard running videos together with a large 1/100 s chronograph stopwatch placed near the trace using cellphone (videos were taken from a distance of about 1.60 m above the ground). Before running, lizards were placed outdoors in direct sunlight to bring their body temperature within the range of 36–38°C, and therefore, decrease the potential influence of body temperature on running speed. The lizards were then placed on the track with their snouts at the starting line, and were simultaneously released after starting the video recording. During the running process, we knocked on the wall of the trace to make the lizards finish the trace running successfully. We measured the distance of each without stopping run (15–103 cm) and the corresponding time, then calculated the speed of each segment and took the maximum speed as the lizard's running speed.

### 2.3 Predation pressure

We produced lizard models using plasticine modelling, with 100 models painted to resemble the darker HSK lizards and 100 painted to resemble the lighter GZ lizards for each transect (see Figure 1). Luminance values were estimated following the protocol described by Tong et al. (2019). We extracted the luminance values of a total of 144 photos using Nikon D7100 digital camera, including 24 photographs for each of dark and light dorsal lizards and their corresponding models, and 24 photographs for each of melanic and non‐melanic habitats. We photographed a Colorchecker Passport white balance card and a 24‐colour card for each photograph, so that we can correct it in the next steps to reduce the error. For all the photographs, we corrected the coloration and white balance using ColorChecker Camera Calibration and Lightroom 5.7. All images were taken in RAW format. Luminance values of lizards/models/substrates were obtained using ImageJ 1.52a. Twenty‐four luminance values acquired from each combination of lizard/model/substrate photographs were averaged. We further compared the average luminance between the dark/light lizards and models, and between melanistic/non‐melanistic substrates and models/lizards. These values are provided as online supplementary table S1. These models were placed on two transects at the adjacent HSK and GZ sites, to test the hypothesis that dark morphology reduces predation at melanistic HSK sites and light morphology reduces predation at non‐melanistic GZ sites. The two different types of models were placed in alternate order along transects, approximately 7 m apart from each other. In each transect, experiments were conducted on different sunny days, with a total of 50 dark and 50 light models placed in melanistic and non‐melanistic habitats, respectively. Bird attacks were assessed from damage to the models (e.g., beak marks), as described in previous studies (Husak et al., 2006; Vervust et al., 2007; Vignieri et al., 2010).

### 2.4 Data analysis

For each individual, the speed (the fastest uninterrupted running distance divided by the time (*t*) between the starting and final running positions of the lizard's snout; m s^−1^) was calculated for the following statistics. All data met the assumption of normality (*p *> .05). Variables were tested for Levene's homogeneity of variance and found that the variances were not significantly heterogeneous (*p *> .05), so we compared the samples by one‐way analysis of variance (ANOVA). Data were presented as means ± SEM, with *p *< .05 considered statistically significant, and all data analyses were performed using SPSS v.20. Because neither sex displayed significant differentiation of running abilities by one‐way analysis of variance (ANOVA) (light lizards: *p =* .729, *F*_1,40_ = 0.122; dark lizards: *p =* .490, *F*_1,84_ = 0.481), we combined sexual data from the same group for the following analyses. The mean values of the speeds of dark or light lizards in non‐melanistic and melanistic habitats are listed in Table 1. Chi‐square analyses were performed to analyze the potential frequency difference of predation attacks on lizard models.

**TABLE 1:** The average running speed (±SEM) of different dorsal color *Phrynocephalus versicolor* in different habitats (m s^−1^)

Habitats	Dorsal color	
	Light color	Dark color
Non‐melanistic habitats	0.558 ± 0.062	0.609 ± 0.053
Melanistic habitats	0.821 ± 0.050	0.626 ± 0.037
samples	21 adults	43 adults

## 3 RESULTS

### 3.1 Speed comparison between melanistic and non‐melanistic populations

The values of the running speed of both groups in different substrates are shown in Table 1. The running speed did not differ significantly between dark lizards on melanistic habitats and light lizards on non‐melanistic habitats (*F*_1, 62_ = 0.063, *p =* .802).

### 3.2 Speed comparison of the same color phenotype between non‐melanistic and melanistic habitats

**FIGURE 2:**
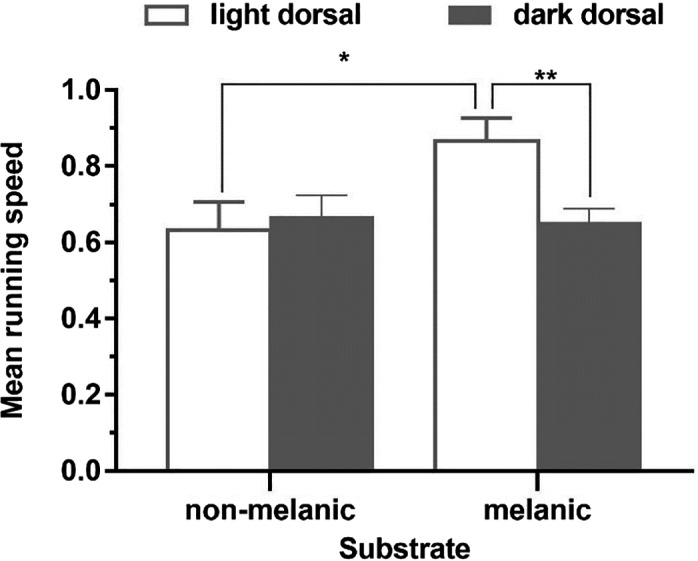
The running speed comparison of light/dark lizards in non‐melanistic/melanistic substrates

The running speed of dark individuals on melanistic substrate were not significantly different than those on non‐melanistic substrate (*F*_1, 84_ = 0.063, *p* = .802). However, the speed of light dorsal lizards was higher on the melanistic substrate than on the non‐melanistic substrate (Figure 2, *F*_1,40_ = 6.115, *p* = .018), which suggests that the light lizards run faster on dorsal‐background mismatch habitats.

### 3.3 Speed comparison of different color phenotypes on the same habitats

On non‐melanistic habitats, there were no significant differences in running speed between dark and light lizards (*F*_1,62_ = 0.143, *p* = .707). On melanistic habitats, the running speed of dark lizards was significantly lower than that of light lizards (Figure 2, *F*_1,62_ = 9.813, *p* = .003). This indicates that running speeds may be connected with crypsis.

### 3.4 Difference in predation attacks

**FIGURE 3:**
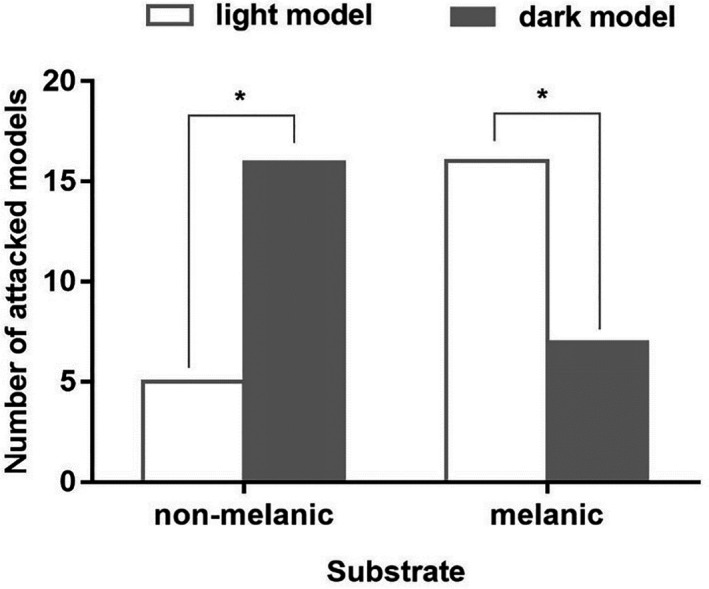
Comparison of anti‐predator pressures: the same dorsal color models between non‐melanistic and melanistic habitats

For luminance values, we found that the luminance was closest between the same color lizards and models, and same color substrates (light lizards: 108.2; light models: 109.7; non‐melanic substrates: 106.6; dark lizards: 62.2; dark models: 61.5; melanic substrates: 62.1). On melanistic HSK, there were a total of 23 models attacked by predators (dark models: 7, light models: 16), with a significant difference between attacks on dark and light models (chi‐square *p* = .046). In contrast, the frequencies of attacked models (dark models: 16, light models: 5) changed and showed significant difference on non‐melanistic GZ habitats (Figure 3, chi‐square *p* = .011). There was no significant difference in model attacks between the HSK and GZ habitats (chi‐square *p* = .749). However, the light and dark lizards both had lower predation pressure on their well‐matching habitat. The chi‐square analyses indicated that the background matched dark or light lizards in HSK and GZ habitats, respectively, were attacked less frequently.

## 4 DISCUSSION

Our results show that dark lizards with better chances of avoiding predators on melanistic habitats have slower anti‐predator running speed than light lizards. The light lizards on the melanistic substrate run faster than those on the non‐melanistic substrate. Our results indicate that camouflage populations might face weaker predation pressure than less background matching populations.

Crypsis and running abilities are major components of the anti‐predation response in lizards, and it has been suggested that dorsal coloration interacts with movement behavior to reduce animal vulnerability to predation (Cooper & Sherbrooke, 2010; Cooper et al., 2008). Some lizards blend their skin color with substrates for concealment (Heideman, 1993). Lizards capable of crypsis can balance the benefits and costs of both choices against predators (Martín et al., 2009). On the melanistic habitats, the dark lizards run slower than the light lizards, indicating that the dark lizards are subject to lower selection pressure of running abilities, possibly due to successful crypsis. Although the running speed may be affected by smoothing of different substrate surfaces (Kolbe et al., 2016), our results indicate that dark camouflage lizards with higher chances of avoiding predators face reduced selection pressure of running speed. Hence, our results suggest that individual running speeds would slow down as the dorsal coloration blends well in their habitats. The lower predation risk due to camouflage could probably reduce predation/anti‐predation requirements through running abilities on the melanistic habitats.

Moreover, background matching is a critical requirement for improving predator or anti‐predator ability in new habitats, and camouflage works when lizard is stationary (Stevens et al., 2011). So, camouflage effect could weaken when the lizard is running. Locomotion does not entirely ‘break’ camouflage (Hall et al., 2013), but it strongly reduces camouflage effectiveness (Baños‐Villalba et al., 2018; Cooper & Sherbrooke, 2010). Background matching functions to avoid the target ever being detected (the first stage of predation), and achieves this simply by increasing the similarity of the target and background (Hall et al., 2013; Webster et al., 2009). Running could decrease the matching of lizards and background (Hall et al., 2013). Therefore, the dark lizards may have evolved low running speed to improve background‐matching ability and decrease the anti‐predation pressure.

Lastly, animal's anti‐predation strategies include camouflage and running capacity, and both strategies need resources, such as proteins and amino acids (Moreno Rueda, 2020; Zamora‐Camacho et al., 2015). The evolution of dark surface for crypsis usually implies the deposition of pigments in the integument and is energetically costly (Moreno Rueda, 2020). Therefore, dark lizards may invest more resource and energy in melanin synthesis to evolve darker surface for better background‐matching at early stage, which causes less investment for later running than light lizards. Therefore, the results suggested that the dark lizards have lower running speed, which due to uneven distribution of energy investment between melanism for early successful crypsis and running.

Interestingly, the running speed of light lizards on the melanistic substrate is higher than that on the non‐melanistic substrate, indicating that light lizards face greater predation pressure in non‐camouflaged environments. We also predicted that this result would be partially correlated with different types of surfaces (Battles et al., 2019; Bergmann et al., 2017; Irschick & Losos, 1999; Kolbe et al., 2016; Li et al., 2011). One possibility is that unsuccessful camouflage on the melanistic substrate may force light lizards to run faster. Another possibility is that the running speed of dark lizards did not differ significantly between melanistic and non‐melanistic substrates. The melanic habitat in HSK is mainly composed of black stones with a rougher surface and greater hardness, while the non‐melanic habitat mainly consists of light‐colored sand and gravel, and the surface is relatively smooth. The former surface being more rigid may make it easier for the light lizards to run (Bergmann et al., 2017; Li et al., 2011). For future studies, we will have an experiment, in which light and dark lizard running performance is tested on melanic and non‐melanic substrate, which have the same composition matrix.

The HSK and nearby Gobi Desert GZ provide an opportunity to study the adaptive significance of traits in an experimental context. Like the White Sand lizard, dark color evolved in the China endemic species *P*. *versicolor* which inhabiting the melanic HSK and is likely a local adaptation to avoid predation (Hardwick et al., 2015). The phenotypic divergence increases with habitat dissimilarity for all species (Rosenblum, 2005). Adaptation can also lead to phenotypic differentiation without genome‐wide divergence if levels of ongoing gene flow are high (Krohn et al., 2019). Studies have shown that the body color variation of *P*. *versicolor* is related to the habitat color, and the color variation of species population is mainly affected by genetic control (evolution) rather than phenotypic plasticity (physiological) (Tong et al., 2016, 2019). The color variation has been linked to mutations in the melanocor‐tin‐1 receptor gene (Mc1r) in lizards (Jin et al., 2020; Laurent et al., 2016; Rosenblum et al., 2010). Whether the color variation of *P*. *versicolor* is related to the variation of MC1R amino acid site remains to be verified by future experiments.

In conclusion, lizards with better camouflage dorsal color might face weaker predation pressure than less matching populations. And we found that the dark lizards have slower running speed maybe because of the successful camouflage by background matching, which meets the need to enhance anti‐predator abilities could decrease the selective pressure on running abilities. Another possible reason is a trade‐off between running and crypsis. The early investment in melanism is energetically costly which caused less investment in later running capacity and reduced the running speed in dark lizards at melanic habitats. We predict that the more investment in camouflage costs and reducing the influence of running on crypsis also enhances anti‐predation ability. Hence, the lower anti‐predation pressure maybe the key reason to weakening the running speed of dark lizards. Moreover, the finding that light lizards could run faster on melanistic substrates than their local, non‐melanistic substrate, is interesting. We suggested that light lizards faced greater running speed selection pressure during the early colonization of non‐concealed environments.

## CONFLICT OF INTEREST

None declared.

## AUTHOR CONTRIBUTION

**Lixia wan:** Conceptualization (equal); Formal analysis (equal); Methodology (equal); Resources (equal); Supervision (equal); Validation (equal); Writing‐original draft (equal). **Zhenxia Liu:** Conceptualization (equal); Formal analysis (equal); Investigation (equal); Methodology (equal); Validation (equal); Writing‐original draft (equal). **Tao Wang:** Investigation (equal). **Minglu Yang:** Investigation (equal). **Jiasheng Li:** Software (equal). **Hui Sun:** Resources (equal). **Chenkai Niu:** Investigation (equal). **Wei Zhao:** Resources (equal). **Yuanting Jin:** Conceptualization (equal); Data curation (equal); Formal analysis (equal); Methodology (lead); Project administration (lead); Resources (equal); Supervision (equal); Writing‐review & editing (lead).

## Supporting information

Table S1

## Data Availability

Data on geographical sampling locations and all measured original running speed data and the attack frequency are shown in FigShare (https://doi.org/10.6084/m9.figshare.14828781). The corresponding authors are responsible for any personal requirements of the materials.
