# Supplementary material for: Camouflage versus running performance as strategies against predation in a lizard inhabiting different habitats
Source: Ecol Evol. 2021 Nov 20;11(23):17409–16. doi: 10.1002/ece3.8374 (PMC8668757; doi:10.1002/ece3.8374)
Supplement: Supplementary file 1 — Table S1 [file ECE3-11-17409-s001.docx]

| Online supplementary Table S1. Sampling information of *Phrynocephalus versicolor* | | | | | |
| --- | --- | --- | --- | --- | --- |
| Sampling localities | Body color | samples | longitude | latitude | altitude |
| one | Dark dorsal | 4males,4females | 95.53°E | 40.95°N | 1,572m |
| two | Light dorsal | 6males,9females | 95.60°E | 40.83°N | 1,415m |
| three | Dark dorsal | 2males,3females | 95.59°E | 40.85°N | 1,452m |
| four | Light dorsal | 2males,4females | 95.61°E | 40.81°N | 1,409m |
| five | Dark dorsal | 5males,13females | 95.44°E | 41.04°N | 1,703m |
| six | Dark dorsal | 2males,10females | 95.39°E | 41.01°N | 1,681m |
|  |  |  |  |  |  |
